# Deep ancestry of Bornean hunter-gatherers supports long-term local ancestry dynamics

**DOI:** 10.1016/j.celrep.2023.113346

**Published:** 2023-11-02

**Authors:** Pradiptajati Kusuma, Murray P. Cox, Graeme Barker, Herawati Sudoyo, J. Stephen Lansing, Guy S. Jacobs

**Affiliations:** 1 Division of Genome Diversity and Diseases, Mochtar Riady Institute for Nanotechnology, Indonesia; 2 Department of Statistics, University of Auckland, New Zealand; 3 School of Natural Sciences, Massey University, Palmerston North, New Zealand; 4 Department of Archaeology, University of Cambridge, Cambridge, UK; 5 Santa Fe Institute, Santa Fe, USA; 6 Complexity Science Hub Vienna, Vienna, Austria

## Abstract

Borneo was a crossroad of ancient dispersals, with some of the earliest Southeast Asian human remains and rock art. The island is home to traditionally hunter-gatherer Punan communities, whose origins, whether of subsistence reversion or long-term foraging, are unclear. The connection between its past and present-day agriculturalist inhabitants, who currently speak Austronesian languages and have composite and complex genetic ancestry, is equally opaque. Here, we analyse the genetic ancestry of the northeast Bornean Punan Batu (who still practice some mobile hunting-gathering), Tubu and Aput. We find deep ancestry connections, with a shared Asian signal outgrouping modern and ancient Austronesian-ancestry proxies, suggesting a time-depth of over 7,500 years. They also largely lack the mainland Southeast Asian signals of agricultural Borneans, who are themselves genetically heterogeneous. Our results support long-term inhabitation of Borneo by some Punan ancestors, and reveal unexpected complexity in the origins and dispersal of Austronesian-expansion-related ancestry.

## Introduction

Borneo, currently the third largest island in the world though historically connected to the mainland during glaciations, was an ancient crossroad for human migrations,^1,2^ and hosts some of the first evidence of Late Pleistocene modern human settlement in Island Southeast Asia (ISEA).^3,4^ The exact timing and routes of early dispersals into Borneo, and the ancestry of those populations, remain unclear, as do the later migration dynamics that lead to the spread of agriculture and Austronesian languages across the island. Essentially, there are two models, of replacement or continuity. In the case of replacement, early occupants related to modern-day Indigenous Australian and New Guinean peoples (though, ancient DNA from nearby Sulawesi might suggest an alternative local deep Asian lineage^5^), were largely replaced by communities speaking Austronesian languages and practising forms of agriculture from ~4,000 years ago^6^ The alternative argues for greater continuity with pre-Austronesian populations and no overwhelming influx of agriculturalist settlers.^7^ Both models tend to make genetic predictions, for primarily external or local ancestry respectively. But untangling the relationship between linguistic diversity, subsistence transition, and genetic ancestry - and implications for population origins and dispersals - is a significant challenge.

Detailed excavations at Niah Cave have been particularly informative, but also raise new questions. The Deep Skull, the earliest known modern human remains in Borneo (~37,000 years ago) has recently been identified as showing local southeast Asian^8^ rather than Australo-Melanesian morphological features,^9^ with potentially profound implications for early dispersal histories. Supplementing this evidence is a rich archaeology documenting subsistence practices, diet, and cultural behaviors and technologies of pre-Neolithic Bornean peoples going back to around 50,000 years ago^4,10^ While an apparent technological transition between late Mesolithic and Neolithic is partially consistent with an influx of Austronesian-technologies, there are clear local complexities and aspects of continuity,^4^ without skeletal morphological changes that might imply population replacement.^11^ The picture of ancient lifeways and their transitions is enriched by data from the Kelabit Highlands, where evidence of human activity based on pollen characteristics in cores stretches to ~4,000 years ago and perhaps 7,000-6,200 years ago. A complex pattern is observed in the millennia following - of foraging, taro and later intensive sago-based subsistence, and stone mound and settlement construction. Rice cultivation appears limited until a very recent transition to rice-based agriculture.^12^ Rock art from the Sangkulirang-Mangkalihat peninsula, Eastern Borneo, gives further insight into ancient lifeways, with a compelling depiction of a small anthropomorph wearing a headdress and holding a spear dated to 13,600 years ago^3^. Overall, the archaeological evidence indicates that subsistence practices and transitions during the Holocene varied markedly.

Genetic evidence concerning both early inhabitants and later migrations is tantalizing but ambiguous. Researchers have identified a multi-layered Asian ancestry in Borneo^13^ that is inconsistent with a “two-layer” model of Australo-Papuan ancestry being replaced by Austronesian-language speakers related to present-day indigenous Taiwanese peoples. The data nevertheless supports strong connections toward Taiwan and the Philippines, more so than with mainland Southeast Asia - as might be expected given an influx of external ancestry.^14^ Ancient DNA and refined analysis of large-scale modern datasets from neighboring islands is pushing the debate forward, with increasing evidence supporting multiple Asian ancestry dispersals predating the presumed Austronesian expansion,^15,16^ as well as a deep, pre-Neolithic mixed ancient-East Asian/Australo-Melanesian ancestry substructure in Sulawesi around 7,000 years ago^5^

The relationship between the early hunter-gathering populations that lived in Borneo and present-day traditional hunter-gatherers of the island - primarily the loosely defined grouping of Punan/Penan^10^ - may be key to resolving subsistence and ancestry transitions. Punan communities are diverse, with varied (Austronesian) linguistic affiliations and subsistence practices (relying variously on sago tree groves as among the Western Penan^12^ and wild tubers, in addition to wild pig, honey, and wide varieties of other fruits, plants, fish and game^17^), deepening uncertainty around their origins and internal relationships. For decades, some scholars have argued that the present Punan might be the descendants of local pre-farming hunter-gatherers^18^ (and, hence, inheritors of Pleistocene Asian ancestry of Borneo), others that they might be the descendants of Austronesian-language speaking farmers who moved into primary forest and in time switched to a hunting and gathering way of life.^19–21^ The generally minimal Australo-Melanesian ancestry in Borneo agriculturalists^13^ as well as in the limited Punan genetic data available^22^ means that only the latter scenario - of full replacement - is consistent with, while not necessitating, proposed Australo-Melanesian Pleistocene ancestry in Borneo.

**Figure 1:**
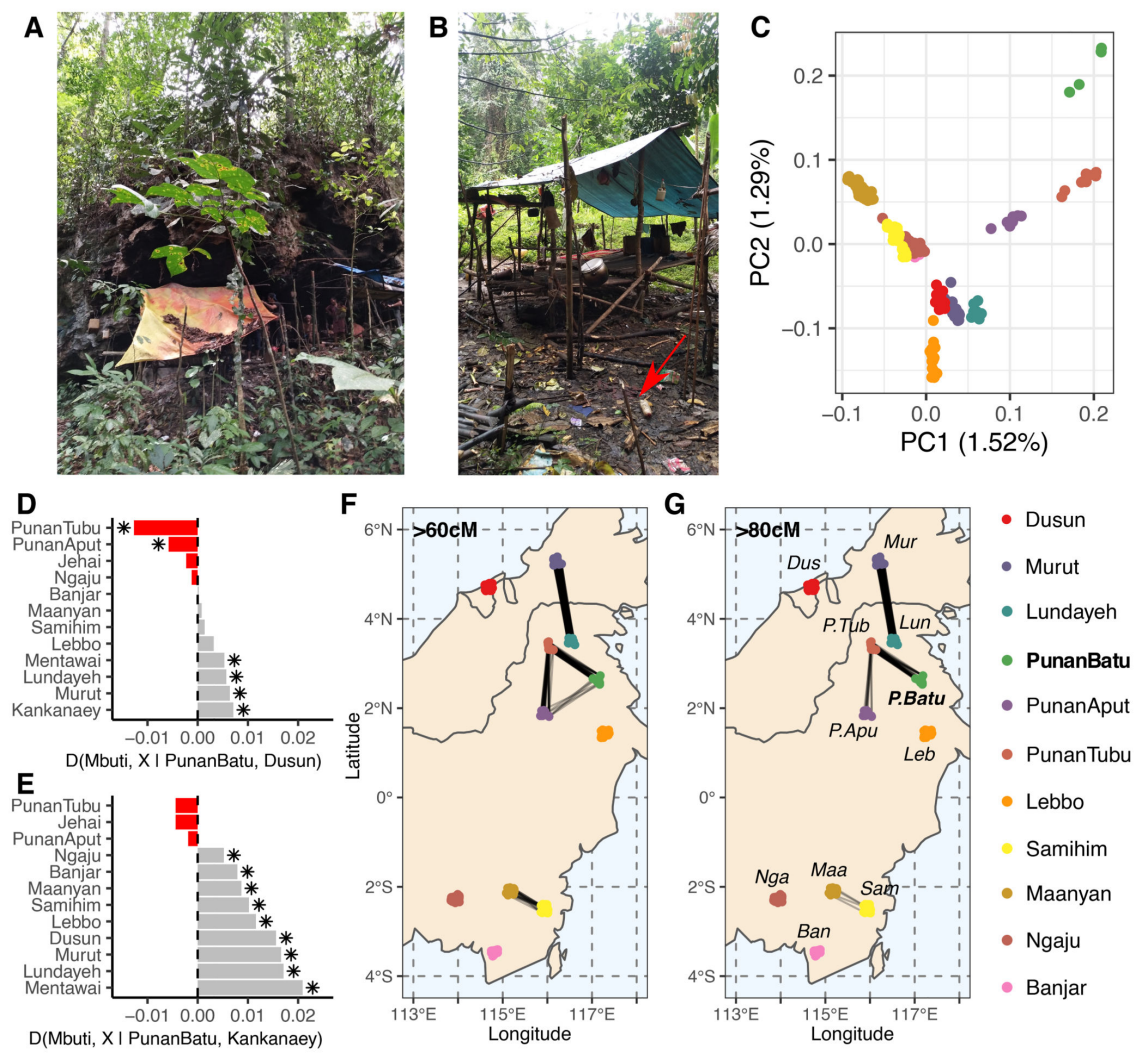
Punan subgroups are closely-related more to each other than to the neighboring agriculturalist groups. A.) A Punan Batu camp under a rock shelter, and B.) near a small stream that was just left by a Punan Batu family (by the time the photo was taken) as they moved camp. They left a message stick (red arrow) indicating the direction of their next camp. (Photo credits: P. Kusuma) C.) A Principal Component Analysis within Borneo populations. D.-E.) D-statistics analyses to observe treeness between test populations (*X* ← Borneans (and Jehai and Mentawai)) with the Punan Batu and the Northeast Borneans Dusun and Kankanaey respectively. F.-G.) Pairwise interpopulation Identity-by-Descent (IBD) with a total length of >60cM and >80cM respectively, as inferred by RefinedIBD. The circles represent individuals in approximate locations as the positions are jittered to avoid overlap.

From mid-2018 to late 2019, we interacted with some of the last active hunter-gatherers in Borneo, the Punan Batu (Cave Punan) of Kalimantan (Indonesian Borneo). The community represents one of a vanishingly small number of groups worldwide still occupying karstic rock shelters (Fig. 1A) and forest camps, and more recently outsider-built riverside shacks, that together form a shifting community network in the forest (Fig. 1B).^22^ While the Punan Batu are referenced in the literature on Borneo hunter-gatherers,^23–25^ they have not been a focus of significant research. They speak a Central Sarawakan Austronesian language,^26^ but they also preserve a unique song language (the Latala language, or *Menirak* (‘to sing’)) that is unrelated to any existing languages in the region.^22^ They actively communicate using message sticks in the forest (Fig. 1B, **red arrow**), hunt on a daily basis, and harvest honey using long lengths of rattan to climb across to sometimes >50m tall smooth-barked “honey trees” (*Koompassia excelsa*) at night. As with other global hunter-gatherers,^27–30^ the lifestyle of the Punan Batu is adapting in the face of ecological disruption, social/political forces and integration into globalized market systems, leading to a rapid transition toward reliance on goods of agricultural origin.

While recent analysis supports long-term genetic isolation from Bornean agriculturalists^22^, the detailed history of the origin of the Punan Batu, and of other more sedentary Punan peoples, remains unresolved. Here, to investigate the genetic history of the Punan and to explore the deeper history of Bornean populations, including their relationship to agricultural populations and implications for the dispersal and transition history of Borneo, we obtained high density genotype data from three Punan groups - the Punan Batu, and two distant resettled Punan communities in North Kalimantan, the Punan Tubu from near Malinau town and the Punan Aput from Long Sule village. These communities are all from northeastern Kalimantan, and represent a focused subset of Punan/Penan diversity − that stretches across central and eastern Borneo − but nevertheless our analyses offer the most detailed investigation into Punan genetic ancestry to date. We study these data in the context of newly generated data from nearby indigenous farmers (the Lundayeh) and a larger dataset representing available high density SNP chip data from northern and southern Bornean agricultural groups as well as the wider region (Table S1).

## Results and Discussions

### Punan as a genetic identity

Summarizing regional genetic variation using Principal Component Analysis (PCA) revealed that all Punan individuals form a tight ancestry cluster that is associated with but distinct from other Bornean groups in our dataset (Fig. S1A). Focussing on diversity within Borneo, there is clear differentiation between the Punan and other indigenous Bornean peoples, including both neighboring agriculturalists (the Lundayeh) and a wide sample of agriculturalist groups from northeastern (NE) and southeastern (SE) Borneo, as well as the mixed-subsistence Lebbo (Fig. 1C). Estimates of individual ancestries using ADMIXTURE v1.3^31^ analysis revealed a Punan-specific ancestral component in the best-supported K = 12 model, most clearly associated with the Punan Batu, but shared by and present in all Punan groups (Fig. S1C). Furthermore, a series of D-statistics^32,33^ in the form of D(Mbuti, *X* | *Y*, NE-Borneo-indigenous-agriculturalist), where *X* and *Y* are Punan groups (Figs. 1D, S1D-L), show negative D-values indicating excess drift sharing between the three Punan groups. Additionally, all non-Punan Borneo groups show more excess drift sharing with Kankanaey compared to the Punan Batu (Fig. 1E). This is not simply a geographical anomaly, the Kankanaey being an indigenous Austronesian-language speaking agricultural population from northern Luzon in the Philippines, but relates importantly to genetic history - the Kankanaey are considered an ‘Austronesian-ancestry proxy’^34^, or a ‘root’ of related ancestry in the region^1,2^, based on their genetic isolation and relationships to modern indigenous Taiwanese groups and ancient DNA (aDNA) samples.

To obtain a broad view on the genetic diversity of the Punan groups, we performed intra- and inter-population pairwise Identity-by-Descent (IBD)^35^ analysis from haplotype data. Longer shared IBD blocks reflect more recent common ancestors while shorter blocks reflect ancient shared ancestry.^36^ Previously, it has been shown that the Punan Batu exhibit unusually long intra-population IBD segments and Runs of Homozygosity (RoH) compared to other populations in Borneo^22^. We confirmed that this excess is observed in the context of the newly represented Punan groups (Fig. S1B), consistent with the small population size of the Punan Batu, which may explain the singular major component in the ADMIXTURE analysis. Substantial total inter-population IBD block sharing is observed between Lun Bawang speakers in Northeast Borneo, the Murut and Lundayeh groups^37^, and between the Southeastern Barito language

speakers in Southeast Borneo, the Ma’anyan and Samihim groups.^38,39^ Among the three Punan groups, all pairs of individuals show long total IBD sharing (Figs. 1F,G), together emphasizing the relatively stronger genetic connections between Punan populations of Northeastern Kalimantan. The ADMIXTURE analysis, D-statistics and IBD-sharing patterns argue that the Punan groups have a signal of shared ancestry, such that ‘Punan’ reflects a genetic as well as a cultural identity, despite their relative geographical isolation.^28,40^

### Punan ancestry is Austronesian-related, but predates “proto-Austronesian”

Ancient connections between agricultural Borneans and MSEA have been proposed on genetic and linguistic grounds.^13,41^ Therefore, to understand the deeper relationship of Punan populations to other Borneans and regional diversity, we first sought to place them on a genetic MSEA-Austronesian proxy axis. This confirms that all island Southeast Asian populations represented, including Borneans, are more closely affiliated with Austronesian-proxy Ami and Kankanaey^34^ than with MSEA groups (Figs. S2A-D). We emphasize that this does not necessarily imply Austronesian-expansion related ancestry in all groups, especially given recent results suggesting Austronesian-related genetic structure in the region predating linguistic and agricultural expansion.^1^ Rather, this simply confirms that Bornean groups are genetically more similar to communities in the islands to the northeast of the region (i.e. Taiwan and the Philippines) than to MSEA populations. To further investigate this signal, we reversed our test to ask whether MSEA communities are more similar to Borneans or to the Kankanaey. This test has the potential to detect hints of weaker genetic links between MSEA and Borneo. This analysis supported the association of MSEA communities to some Bornean groups, emphasizing regional complexity in a broadly Kankanaey/Ami-related genetic context (Figs. S2E-J).

To explicitly test the relationship between Punan and Austronesian/Austroasiatic-speaking populations, we use qpGraph.^32,42^ qpGraph builds bifurcating population trees with statistically significant admixture nodes to capture past divergence, drift and mixing of populations. We incorporated data from ancient East Asian individuals,^5,16,43–45^ including those considered “Proto-Austronesians” (an early Neolithic Liangdao2 (~7,500 yBP) individual from Liang Island, and a mid-Neolithic Suogang-B1 (~4,500 yBP) individual from Fenghui Island, Southern China Sea, here referred as “coastal southern East Asia ancestry” (cSEA)),^16^ to situate the Punan Batu genetic background within the East Asian/Austronesian ancestry spectrum. If the Punan Batu are descendants of Austronesian-speaking migrants who reverted to hunting and gathering,^20,21^ a recent Austronesian-ancestry signal, demonstrated by a close derived ancestry association with the Kankanaey, Ami and especially ancient cSEA ancestry, should be present in their genomes.

**Figure 2:**
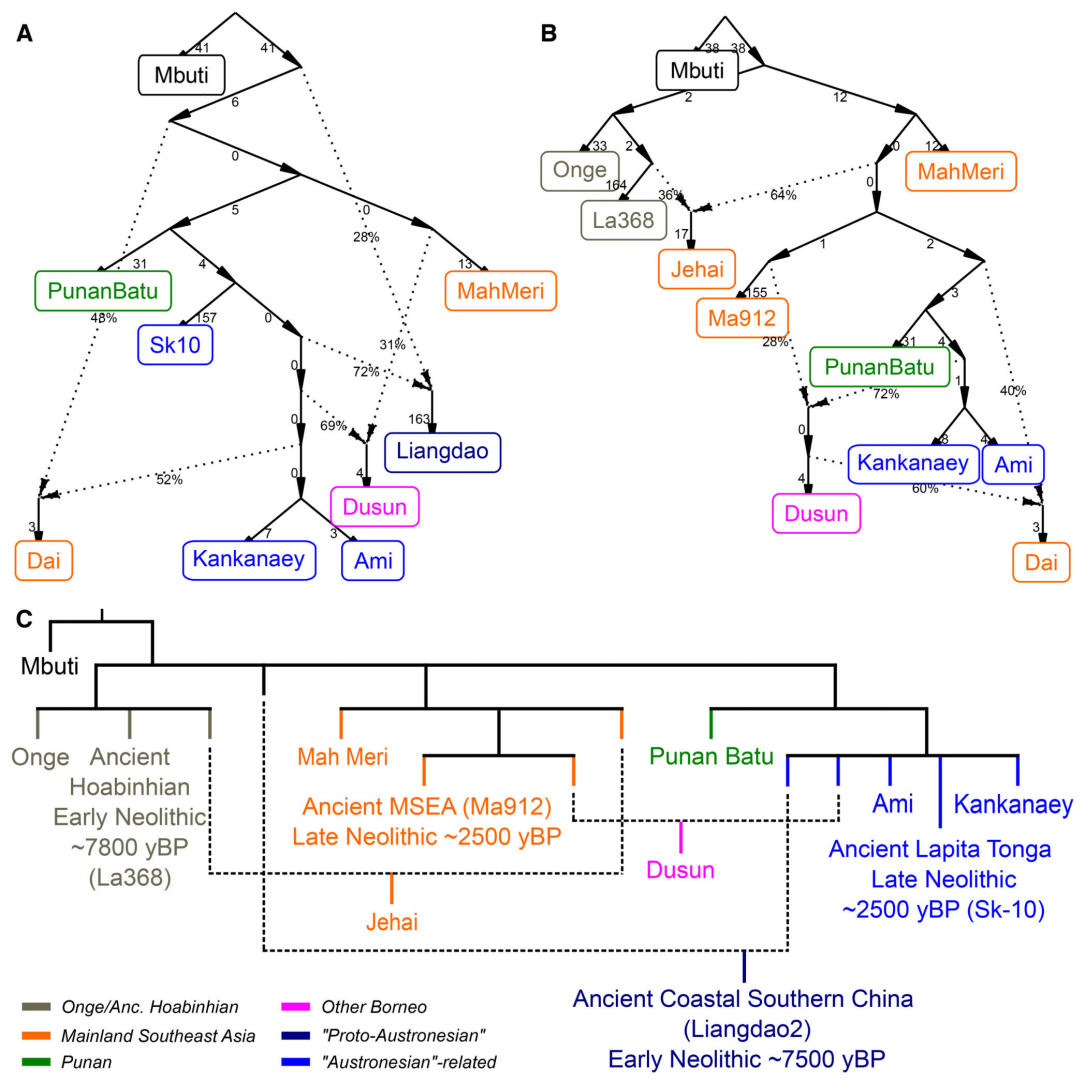
The qpGraph analyses show deep divergence of Punan split. A.) A qpGraph analysis (worst |*Z*| = 2.681) of Punan Batu and Northeast Bornean (Dusun) population history, combining present day populations with ancient East Asian (Liangdao2) and Lapita individuals (Sk-10), and Punan Batu predates the split, of which the drift contributes to the Early Coastal Southern East Asia / pre-Austronesian lineage. B.) Another qpGraph analysis (worst |*Z*| = 2.625) with Early (La368) and Late Neolithic (Ma912) individuals from Mainland Southeast Asia confirming Punan Batu placement before the Kankanaey-Ami cluster and there is also no gene-flow from Austronesian-related ancestry to Punan Batu. Numbers near arrows represent drift unit, and percentages represent admixture proportion. C.) A schematic of population history reconstruction inferred from the qpGraph analyses.

The qpGraph analysis (Figs. 2A, S3A) shows that the Punan Batu are placed deep within a split before the cSEA (represented by an early Neolithic Liangdao2 individual, ~7,500 yBP).^16^ Other aDNA samples confirm this depth. Analysis of mid-Neolithic Austronesian-proxy individual (Suogang-B1 (~4,500 yBP) from Fenghui Island, Southern China Sea)^16^ (Figs. S4) and the ancient Austronesian Lapita individual in the Southwest Pacific (Sk-10 (~2,500 yBP) from Tonga)^43^ (Fig. 2A) shows that both share substantial derived drift with the Kankanaey relative to the Punan Batu. Interestingly, the cSEA ancestry among the Dusun forms part of the Ami/Kankanaey/Sk-10 ‘radiation’ (Fig. 2A). Together, these samples offer strong support for the overall depth and complexity of cSEA-related ancestry, and the placement of the Punan Batu within it.

Available aDNA from Borneo is limited to two genetically similar historical (~410 yBP) agricultural individuals, Ma554 and Ma555, from Sabah, northeast Borneo^15^, and to understand recent Bornean ancestry dynamics we incorporated Ma554 into our analyses. Using D-statistics, we find that Ma554 has close affinity to the Austronesian-proxy Kankanaey and Ami (Figs. S5A,B) and - surprisingly, to a lesser rather than greater extent - to agricultural Northeast Borneans from the same geographical area as the sample, i.e. the Lundayeh and Murut, consistent with qpGraph analysis (Figs. S5C-D). That both Ma554 and regional modern Borneans are divergent from the Punan Batu - but in slightly different ways - supports the overall picture of long-term separation between the northeast Bornean Punan and agricultural communities.

**Figure 3:**
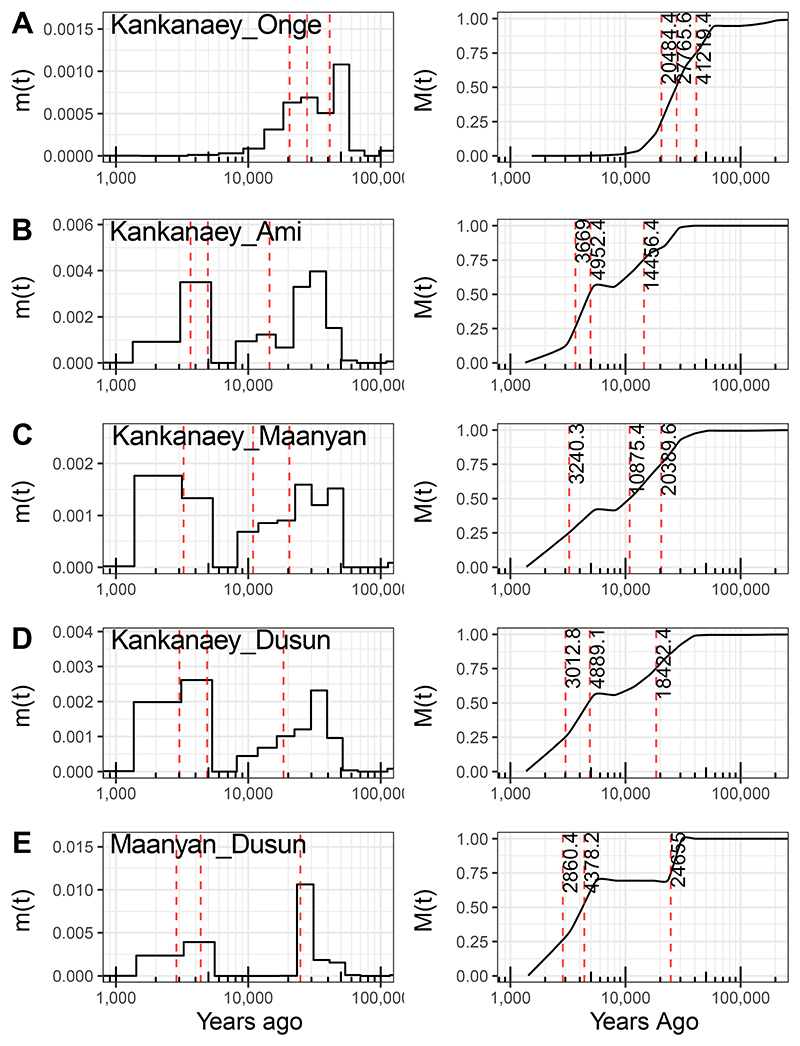
Split times estimation using MSMC-IM. The analyses are conducted between Kankanaey and A) Onge, B) Ami, C) Maanyan, and D) Dusun, also E) between Maanyan and Dusun. Panels on the left show the distribution of haplotype coalescence times, with the cumulative distribution on the right. Note that multimodal distributions suggest multi-phase migration processes - for example, the more recent mode observed in the Kankanaey-Ami and Kankanaey-Dusun analyses possibly reflects shared ancestry due to the Austronesian expansion, with a peak 5,000-3,000 years ago as expected in the dominant archaeological/linguistic model. Deeper modes suggest more ancient contacts postdating initial dispersals to the region.

High coverage modern genomes offer a complementary route to assessing divergence times and dynamics using haplotype analysis. We first assessed the time-scale of Onge-Kankanaey divergence, providing an upper bound for the emergence of the Punan clade. We used MSMC-IM to estimate the distribution of haplotype coalescence reflecting the structure and timing of shared ancestry,^46^ finding a median split of 27,700 years ago (20,400-41,200 years ago MSMC-IM IQR) (Fig. 3A). Similarly, a lower-bound is provided by the Kankanaey-Ami split; a multimodal distribution reflecting multiple contact processes is inferred, with a median split of 4,900 y.a (3,600-14,000 years ago MSMC-IM IQR; Fig. 3B). Interestingly, the median date corresponds quite closely with the onset of the Austronesian expansion in an Out of Taiwan model,^6^ but with a composite migration signal over time that generates multiple phases of population divergence indicative of greater complexity. Finally, we directly dated the Kankanaey-related contribution to Borneo based on Dusun-Kankanaey analysis, finding a similar multimodal distribution and early median date (4,800 years ago [3,000-18,000 years ago]; Fig. 3D). Importantly, based on both aDNA evidence (the placement of 7,500 years ago old Liangdao2 in the ancestry graphs) and divergence-time inference, the Punan Batu genetic ancestry pre-dates the arrival of potentially Austronesian-expansion related ancestry into Borneo.

We finally use the breadth of available East Asian and Southeast Asian ancient DNA samples in D-statistics to investigate the history of endogenous ancestry in Borneo (Fig. S6). All ancient East Asian samples - including both northern inland (e.g. Yumin) and coastal East Asians (e.g. Boshan) - show stronger affinity towards Kankanaey, rather than Punan Batu, a pattern that is partially replicated when considering Bornean ancestry more broadly. Intriguingly, both a Late Neolithic (~2,100 yBP) northern highland Sumatran (Gayo) sample,^15^ In662, and a late-Neolithic (~2,500 yBP) MSEA sample,^15^ Ma912, show either significant or tentative associations to the majority of Bornean populations over the Kankanaey. Our analyses therefore again confirm the long-term divergence of Bornean and Kankanaey-related ancestry, and the time-depth of endogenous ancestry dynamics.

### The ancient MSEA connection with the people of Borneo

To better investigate the MSEA connections suggested by Ma912, we performed further D-statistic tests (Figs. S7A-C and qpGraph/TreeMix reconstructions (Figs. 2B, S8-9). We find significant connections between Ma912 and Bornean populations relative to the Jehai, but generally balanced D statistics when considering Ma912 on an axis of Bornean and Mlabri/Mah Meri ancestry (Figs. S7A-C), consistent with the placement of Ma912 in the qpGraph (Fig. 2B) and suggesting links between some, but not all, MSEA ancestries and Bornean populations. Potentially different connections between Ma912 and various Bornean populations (Figs. S6-S7) lead us to construct additional qpGraphs focussing on both northeast and southeast Bornean populations (Figs. S8-S9), confirming connections to both MSEA and coastal southern East Asian ancestries.

Incorporating Ma912 into these graphs also allows us to refine our understanding of MSEA-related and Austronesian-related ancestry in the non-Punan Bornean populations. For example, the ~28% MSEA-related contribution to the Northeast Bornean Dusun and Lundayeh are related to the Ma912 (Figs. 2B, S8A, S9A). Reconstructions of the MSEA component in the Southern Bornean groups (Maanyan and Ngaju) are also Ma912-related (Figs. S8B-C, S9B-C). We note that both proposed Austroasiatic linguistic substructure in Borneo,^41,47^ and the linguistic context of proposed Greater North Bornean languages in MSEA^48^ are consistent with complex and long-term Borneo-MSEA interactions.

Interestingly, other Punan groups, the Aput and Tubu, exhibit minor admixture from MSEA ancestry as well (Figs. S6-S7), whether directly or via other Bornean groups, despite primarily deriving ancestry from the un-admixed Punan Batu (Figs. S8D, S9D).

In sum, the Punan Batu differ from neighboring agriculturalists in two key ways. First, our qpGraph analysis shows that their core ancestry neither emerges from the Austronesian-expansion related multifurcation, nor is derived from MSEA groups. Instead, the Punan Batu are phylogenetically placed between the two, outgrouping modern proxies of Austronesian ancestry and lacking derived drift observed in relevant ancient samples (Sk-10, Liangdao2 and Suogang-B1). Secondly, unlike all other Bornean groups tested, we do not find a composite mix of Austronesian-related and MSEA-related ancestry. The first result strongly argues that the ancestors of these Punan groups were not simply Austronesian seafarers, while the second argues for a degree of long-term isolation of some Punan from Bornean agriculturalists. The Punan therefore show a separate settlement history from ancient MSEA and (Proto-)Austronesian/cSEA in Borneo, and distinct ancestry that is partially independent within broader Bornean complexity. Our results indicate considerable time depth of genetic isolation, and confirm that these NE Kalimantan Punan groups are not a recent subsistence reversion of neighboring indigenous farmers.^2,14^

### The presence of Papuan-related ancestry in Borneo

**Figure 4:**
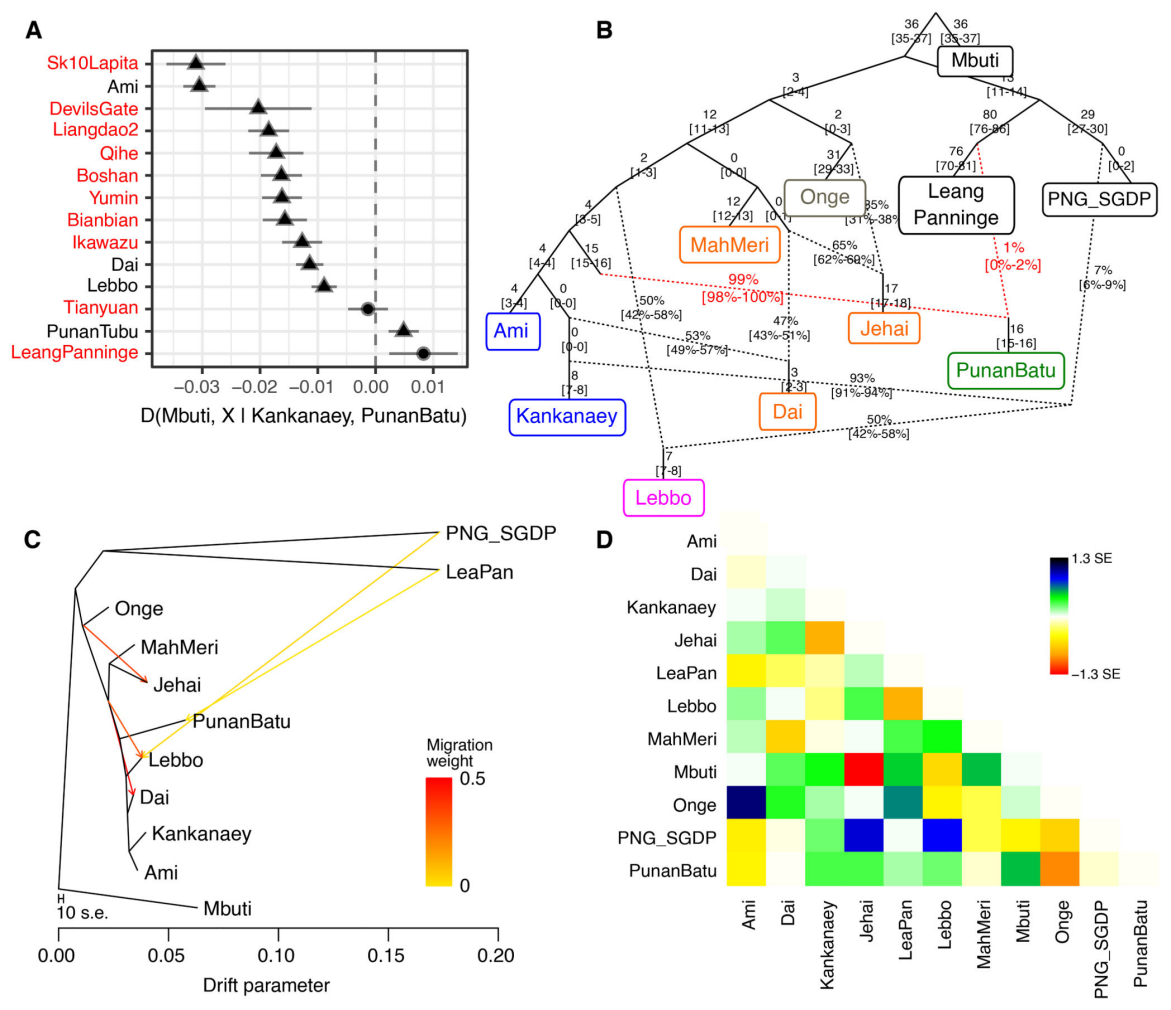
Formal admixture tests involving the ancient Leang Panninge data. A.) A D-statistic analysis shows a tentative affiliation between ancient Leang Panninge individual with the Punan Batu (● = Z-score < |2|; ▲ = Z-score > |2|). B.) A qpGraph analysis (worst |*Z*| = 2.143) confirms the possible weak link from Leang Panninge to Punan Batu (0-2%), which is also confirmed by C.) and D.) TreeMix analysis, exhibiting migration nodes from Papuan to Lebbo (4.3%), and from the ancient Leang Panninge individual to Punan Batu (1.05%).

The depth of Punan Batu ancestry raises immediate questions regarding its relationship with other ancient hunter-gatherers within Island Southeast Asia. To investigate this we draw on the recently reported pre-Holocene ancient DNA sample from Leang Panninge^5^ (South Sulawesi), which shows composite Papuan/ancient Asian-related (Tianyuan/Onge) ancestry. We observe a tentative signal of shared ancestry between the Punan Batu and Leang Panninge using D-statistics (Fig. 4A), which resolves as a weak admixture node in our qpGraphs (Fig. 4B; ~1% supported in best-fitting graph, with 0-2.1% confidence interval). Given the limited nature of this signal we sought to confirm it using TreeMix (Fig. 4C) and again detect a weak connection between the Punan Batu and the Leang Panninge sample.

Noting that Leang Panninge is itself admixed, incorporating Papuan-related ancestry, we constructed our graphs to also include both the Lebbo population from eastern Borneo, who have a well-documented, though as yet unexplained, Papuan ancestry component. We confirm that the Lebbo admixture signal is not connected to the Leang Panninge sample or to the Punan Batu (Figs. 4B,C). ALDER^49^ analysis estimates the admixture date to be around 85.5 generations ago (~2,480 years ago (3,360-1,600 years ago), with 1 generation ≈ 29 years), generally consistent with previous results.^50^ Thus, while not yet conclusive, multiple methods suggest a weak ancestry connection to Leang Panninge observed in the Punan Batu, independent of known Papuan-related ancestry in the region.

### Limitations of the study

Our study presents strong evidence for shared genetic ancestry among Punan communities, rejecting historic suggestions that they were Austronesian-expansion derived farmers who underwent recent agri-cultural reversions. Although the study includes several northeast Bornean Punan communities and a diverse agricultural Bornean comparative dataset, inclusion of more Punan/Penan groups would better determine the degree to which these communities are related and their wider history. While our high-density genotyping approach is generally well suited for demographic reconstruction, whole-genome sequencing and larger sample sizes would support inference by revealing patterns of local low-frequency variant sharing. More significantly, full resolution of genetic demography of the region, as well as detailed dynamics and ancestry associations of subsistence change, would ideally require further archaeological work and ancient DNA data, spanning a diversity of subsistence practices and time-scales, both within Borneo and island Southeast Asian more broadly. Given poor DNA preservation in tropical regions and relatively limited archaeological data over key time horizons, this is likely long-term research effort. Nevertheless, our results are inconsistent with simple models of ancestral origins and subsistence reversion, indicating long-term habitation of Borneo by the ancestors of some Punan peoples, and supporting complexity in the ‘Austronesian expansion’ story in western Indonesia.

## Conclusion

Our analyses combine available ancient and modern genetic datasets to reveal the complexity of ancestry among Bornean peoples, including multiple Punan populations. We confirm and refine evidence of the diverse and composite MSEA, Punan-related, and Austronesian-related ancestry of agricultural Bornean groups. Most importantly, our work provides significant insights into the ancestry of Punan communities from northeast Borneo. We confirm that Punan ancestry is neither Austronesian-derived, nor mirrors that of Bornean agriculturalists - thus arguing strongly against a model in which modern Punan groups underwent subsistence reversions from either agricultural or mixed-strategy early-Austronesian pioneers, or from local Bornean groups. While ancestry is not necessarily a marker of subsistence practices, the cumulative evidence of widely shared cultural practices (e.g. message sticks), well-established archaeological evidence of hunter-gatherer groups in Borneo stretching from ~50,000 years to recent millennia, and ancestry signals couple to strongly argue for considerable time depth of hunting and gathering, no doubt in various manifestations in different places and times,^51^ among the ancestors of many if not all present-day Punan.

**Figure 5:**
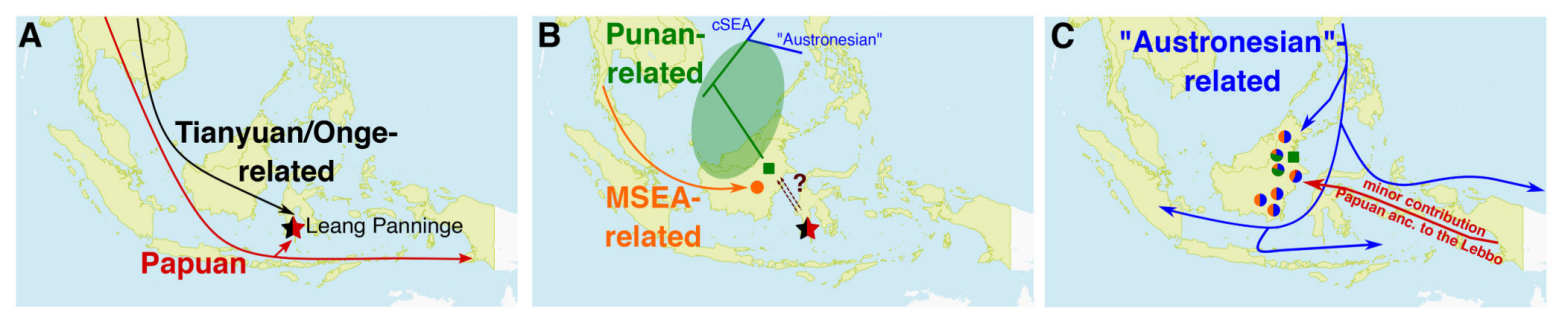
Schematic summary of a proposed population settlement in the region. A.) Initial occupation of Sunda and Sahul by ancestry related to modern New Guinean and Australian Aboriginal populations, followed by deep mainland Asian (Tianyuan- or Onge-related) ancestry as evidenced by the Liang Panninge sample. B.) Dispersals of ancestries associated with ancient Mainland Southeast Asian and ancestral Punan-related components predating the coastal South Chinese, and hence Austronesian-related, ancestries. C.) Austronesian expansion leading to Austronesian (Ami- and Kankanaey-related) ancestry observed in NE and SE Borneans, and subsequent specific Papuan ancestry admixture observed in the Lebbo population in East Borneo.

Our findings support layered regional processes of initial colonisation (Fig. 5A), the dispersal of MSEA-related to Borneo and emergence of endogenous Punan-related ancestry (Fig. 5B), and later ancestry influx that may reflect Austronesian-related dispersal or contact (Fig. 5C), combining to generate the complex and diverse ancestry of Bornean communities. The ancient, pre-Austronesian signal in the Punan Batu is particularly informative. At a minimum, this suggests ancient connections from Borneo to the northeast and ancient proto-Austronesian coastal southern East Asian ancestry (Fig. 5B), coupled with significant long-term isolation. Whether we are capturing one spoke of an interconnected and distributed pre-Austronesian ‘coastal southern East Asian’ wheel (e.g. considering also other recent results from the Phillipines^1,2^), likely with a significant though not impermeable Wallacean boundary^5^, or whether Punan ancestors could have been a source involved in founding coastal south East Asian ancestry before the later spread of agriculture and Austronesian languages remains unclear. Further work - as mentioned above, ideally aDNA from Borneo and the wider region, spanning the birth and dispersal of the Austronesian language family - is required to disentangle these possibilities. In the meantime, our analyses demonstrate the deep ancestry complexity of Borneo, and the unique history of the Punan within this.

## STAR Methods

### Key Resource Table

**Table:** 

Reagent or Resource	Source	Identifier
**Biological Samples**		
see Table S1	Eijkman Institute for Molecular Biology, Indonesia (currently, Mochtar Riady Institute for Nanotechnology, Indonesia)	N/A
**Deposited Data**		
Genotype files	European Genome-Phenome Archive (EGA; https://www.ebi.ac.uk/ega/home)	Accession number: EGAS00001004471
Comparative modern genotype data	Multiple data resources from publications	Table S1
Comparative ancient genotype data	Allen Ancient DNA Resource (AADR)	https://reich.hms.harvard.edu/allen-ancient-dna-resource-aadr-downloadable-genotypes-present-day-and-ancient-dna-data
Comparative ancient genotype data from Leang Panninge	European Nucleotide Archive	Accession number: PRJEB43715
**Software and Algorithms**		
PLINK v1.9	Chang et al. 2015	https://www.cog-genomics.org/plink/
KING	Manichaikul et al. 2010	https://www.kingrelatedness.com/
EAGLE v2.4.1	Loh et al. 2016	https://alkesgroup.broadinstitute.org/Eagle/
SNPRelate	Zheng et al. 2012	https://bioconductor.org/packages/release/bioc/html/SNPRelate.html
ADMIXTURE v1.3	Alexander et al. 2009	https://dalexander.github.io/admixture/
CLUMPP v1.1.2	Jakobsson et al. 2007	https://rosenberglab.stanford.edu/clumpp.html
Refined IBD	Browning and Browning 2013	https://faculty.washington.edu/browning/refined-ibd.html
ADMIXTOOLS v7.0	Patterson et al. 2012	https://github.com/DReichLab/AdmixTools
TreeMix v1.3	Pickrell et al. 2012	https://bitbucket.org/nygcresearch/treemix/wiki/Home
ADMIXTOOLS2	Maier et al. 2023	https://github.com/uqrmaie1/admixtools
MSMC2	Schiffles et al. 2014	https://github.com/stschiff/msmc2
MSMC-IM	Wang et al. 2020	https://github.com/wangke16/MSMC-IM

## Resource Availability

### Lead contact

- Further information and requests for resources should be directed to Guy S Jacobs (gsj22@cam.ac.uk)

### Materials availability

- No new materials were generated in this project.

## Experimental Models and Study Participant Details

### Human Research Participants and Ethic Approval

All samples were obtained from adult human research participants. For full information about the new and published samples used in this study, refer to Table S1 − Sample and combined dataset list.

Biological sampling was conducted by Indonesian researchers with the assistance of Indonesian Public Health clinic staff, following protocols for the protection of human subjects established by the Eijkman Institute Research Ethics Commission. All samples were collected with informed consent. Collection, use of samples, and biological data analysis were approved by the Eijkman Institute Research Ethics Commission, Indonesia (EIREC#122, 2018). Ethnographic observation, filming, social and linguistic surveys were approved by the Institutional Review Board of the University of New Mexico (IRBNet ID: [1290975-4]).

### Biological Samples

Volunteers were surveyed for language affiliation, current residence or location of camps/rock shelter for the Punan Batu, familial birthplaces and a short genealogy of three generations to ensure regional and ethnolinguistic ancestry. A total of 40 samples were analyzed from 3 Punan communities (Punan Batu from Bulungan Regency, North Kalimantan Province (n=12); Punan Aput from Long Sule village, Malinau Regency, North Kalimantan Province (n=10); and Punan Tubu from a Sembuak resettlement village, Malinau Regency, North Kalimantan Province (n=10)); and 1 non-Punan agricultural community (Lundayeh from Pulau Sapi village, Malinau Regency, North Kalimantan Province (n=8)) (Fig. 1F-G; Table S1). All samples in this study were from male research participants in order to be able to cover both sex chromosomes. The age range was between 20-64 years old. We also added DNA samples from Mentawai, West Sumatra (n=18) and Korowai, Mappi District, West Papua (n=5) as comparative populations. Samples are all from blood, and the DNA was extracted using Gentra Puregene Blood Kit (Qiagen, USA) following the manufacturer’s protocol. All laboratory work was conducted at the Eijkman Institute for Molecular Biology, Jakarta, Indonesia.

## Method Details

### Genotyping and Dataset Integration

The samples were genotyped using the Illumina Omni 2.5 array, resulting in 2.4 million variants typed across the genome, of which 2.3 million are autosomal variants. A comparative data set was built from 50 worldwide populations comprising an additional 641 individuals (Table S1). This dataset incorporates the major high-density SNP chip genetic data currently available for Bornean populations - the agriculturalist Dusun and Murut from northern Borneo, the Lebbo people from eastern Kalimantan, and several agricultural groups from southeast Borneo (the Ngaju Dayak, Ma’anyan, Samihim and Banjar). Among these groups, the Lebbo, though currently agricultural, historically followed hunter-gatherer and horticulturalist subsistence.^23^ Data quality controls were performed using PLINK v1.9^52^: (i) to avoid close relatives, relatedness was measured between all pairs of individuals within each population using an identity-by-descent (IBD) estimation (upper threshold: 0.25 i.e. second-degree relatives) and confirmed using KING (upper threshold: 0.084),^53^ leading us to remove 4 Punan Batu individuals; (ii) SNPs that failed the Hardy–Weinberg exact test (P < 10^−5^) in each population were excluded; (iii) samples with an overall call rate < 0.99 and individual SNPs with missing rates < 0.05 across all samples in each population were excluded. Genotypes were phased with EAGLE v2.4.1^54^ using the Indonesian Genome Diversity Panel pilot (IGDP)^55^ phased data as a reference panel, which covers more variants in island Southeast Asia and, importantly, Indonesian populations. For certain analyses, we applied Linkage Disequilibrium (LD) SNP pruning using PLINK v1.9. Pruning was performed in 50-SNP sliding windows with a step size of 5 SNPs, and SNPs with r^2^ > 0.2 were removed. The final dataset (before pruning) contains 227,102 SNPs. For certain analyses, we incorporated Mbuti, Onge, and Papuan individuals from the Simons Genome Diversity Panel (SGDP),^56^ of the original HGDP data,^57^ to preserve higher density SNP data, with ancient Mainland Southeast Asian and Bornean samples additionally included, especially for for TreeMix and qpGraph analyses. A series of allele- and haplotype-based analyses were performed to disentangle the genetic history of the Punan.

## Quantification and Statistical Analyses

### Observing Population Structure and Diversity

Population structure was evaluated using a suite of different programs/algorithms to make robust inferences. Principal Component Analyses were built using the SNPRelate R package^58^ after LD SNP pruning, applying a stricter PI_HAT threshold (PI_HAT < 0.1) and without removing any outliers. The regional PCA showed that Punan formed a tight cluster near other Borneo groups (Fig. S1). We then built a PCA of Borneo groups - Dusun and Murut (from Brunei and Sabah (Northeast Borneo), respectively), Lebbo’ (from East Borneo), Ma’anyan and Samihim (Southeast Barito language speakers from Southeast Borneo), Ngaju (major group from South Borneo; speakers of the ritual Sangiang language), Banjar (Malay speakers from Southeast Borneo), as well as Punan and Lundayeh (neighboring agriculturalists). Punan individuals clustered with their respective groups, clearly distinguishable from the neighboring agriculturalists. The Punan Aput are pulled towards the Punan Tubu pole, while the indigenous agriculturalist Lundayeh individuals are grouped with the other Northeastern Borneans agricultural groups (Murut and Dusun) (Fig. 1C). Punan Batu served as an outlier and drove both PC1 and PC2, likely reflecting drift and/or inbreeding in the population, as also shown in IBD and ADMIXTURE analyses below.

ADMIXTURE v1.3^31^ was used to observe population structure by classifying individuals into K clusters based on genetic similarity using maximized likelihood with high-dimensional optimisation. Ten randomly seeded runs were performed for each number of ancestral populations (K = 2–20), and the results within each K were summarized with CLUMPP v1.1.2^59^ (Fig. S1C). Cluster K = 12 was shown to have the lowest cross-validation error value, and was therefore chosen as the best model describing the dataset. At K = 12, Punan Batu individuals have one ancestral component, most likely due to genetic drift. This component was also detected in other Punan groups (Punan Aput ~ 7%; Punan Tubu ~ 9-10%), but it was negligible among non-Punan populations.

To obtain a broad view on the genetic diversity of the Punan groups, we performed intra- and inter-population pairwise Identity-by-Descent (IBD) analysis from haplotype data. Haplotype sharing using the Refined IBD^35^ was computed to estimate the total number of shared genetic fragments (logarithm of odds ratio (LOD) > 3) between each pair of individuals. The number of shared IBD segments > 2 cM was used to compute the distribution between each Punan group and other populations from the dataset. Meanwhile, the Runs of Homozygosity (ROH) analysis was performed in PLINK v1.9^52^ from the linkage-disequilibrium-pruned dataset. The Punan Batu share long IBD chunks with other Punan groups, with considerable sharing of Punan IBD chunks among all Punan groups (Fig. 1F-G). This implies the sharing of recent common ancestors, despite marriage not being reported between these groups and their use of different languages. We also confirmed high total runs of homozygosity in the Punan Batu and other regional traditional hunter-gatherers, relative to regional agriculturalists (Fig. S1B).

### Testing the “Tree-ness” by Involving Contemporary Populations and Ancient Individual Data

We used a range of D-statistics calculated using ADMIXTOOLS v7.0^32^, to interrogate regional ancestry in the context of modern and ancient samples. D-statistics is a test of treeness based on drift/allele frequency sharing between populations, on the form of D(Outgroup, *X* | *A, B*). When the D values are significantly positive (Z > 3), it is indicative of gene flow occurred between the “*X*” and “*B*” group (relative to “*A*” group), conversely, when negative (Z < -3), it is indicative of gene flow occurred between the “X” and “*A*” group (relative to “*B*” group). When the values are non-significant the tree is considered balanced. To investigate Punan Batu ancestry, we assigned African Mbuti as the outgroup and “*X*” as all Borneans populations in the dataset, while “*A*” and “*B*” are the Punan groups (Punan Batu, Punan Tubu, and Punan Aput) and the neighboring indigenous agricultural groups in Northeast Borneo (Dusun, Murut, and Lundayeh) respectively. Interestingly, all Borneans show stronger affinity to Kankanaey, except the Punan Tubu and Punan Aput who show affinity to the Punan Batu (Figs. 1D-E, S1D-L). Together with the ADMIXTURE and IBD analysis, this result suggests that ‘Punan’ is not only a cultural identity,^28^ but that they also share common ancestry.

We also performed D-statistics to test genetic affinity of the Punan groups and other Borneans in the dataset to either Austronesian-proxy or Mainland Southeast Asian (MSEA) populations (Figs. S2). The “*A*” and “*B*” are the references for MSEA- (Mlabri or Jehai) and Austronesian (Kankanaey or Ami)-related ancestry respectively. We found that most Borneans have stronger affinity towards modern Austronesian-related ancestry compared to MSEA-related ancestry, including the Punan groups (Fig. S5). In contrast, the indigenous Aslian groups from Peninsular Malaysia showed affinity towards MSEA, as expected from their geographical and linguistic connections with MSEA. We included ancient Proto-Austronesian (ancient Mid Neolithic coastal Southern East Asia (Suogang-B1)^16^, an ancient historical Ma554 individual (~410 yBP, from Sabah, Northeast Borneo),^15^ as well as other ancient individuals in East Asia in the analysis (Fig. S4, S6). All ancient East Asians showed their affinity towards Austronesian Kankanaey/Ami relative to the Borneans (Fig. S6). While supporting the evidence of influence from Austronesian-related ancestry in the ancient DNA samples in the region, the Austronesian-related genetic trace is evident in the contemporary NE Bornean agricultural groups, but not in the Punan. Further D-statistics involving an ancient MSEA Late Neolithic Ma912 individual (~2,500 yBP from Kelantan, Malaysia) and an ancient MSEA Late Neolithic In662 individual (~2,150 yBP from Gayo highland, North Sumatra, Indonesia) showed hints of gene flows from the ancient, but not from the contemporary, MSEA ancestry to Borneans in our dataset (Fig. S7). The evidence is quite pronounced in almost all Bornean groups, except the Punan Batu and Lebbo, which appeared to be weak (i.e. the Z-scores < 3).

### Testing the Admixture Graph Models

To make ancestral inferences beyond bi-ancestry D-statistics, we used TreeMix v1.3^60^ analysis. This method constructs a tree based on a drift parameter, before inferring migration nodes on the tree based on the ancestry covariance matrix. We set Mbuti population as the root, used blocks of 100 SNPs to account for linkage disequilibrium (−k100), and allowed migration edges (m) to be added sequentially until the model explained 99% of the variance (Figs. S3-S5, S9). We also used qpGraph to assess the fit of admixture graph models to allele frequency correlation patterns as measured by f2-, f3-, and f4-statistics.^32,42^ We performed extensive automatic optimization of qpGraph runs using *find_graph()* function in ADMIXTOOLS2 R package.^61^ We performed qpGraph fittings with 2 - 5 admixture events using African Mbuti as the outgroup population. The algorithm constructed 10 graphs per iteration for 100 iterations (generations), with new graphs constructed based on the best-fitting graphs in the previous generation. We retained the graph with the best score across all iterations for the 2 - 5 admixture event models, and when possible, present the graph with fewest admixture events that still had no individual f4-statistics |Z score| above 3.

The result demonstrates that the Punan split earlier than the “Proto Austronesian”/coastal South East Asia lineage, here proxied by the ancient Liangdao-2. Punan groups share a recent common ancestor which was derived from a split between Early MSEA and coastal southern East Asia/pre-Austronesian lineage (Fig. 2A-B, S6). There is no gene flow from neighboring indigenous agricultural groups to the Punan Batu Sajau, or from the Austronesian Kankanaey. Minor gene-flow from Early MSEA is potentially also observed in the Punan Aput and Punan Tubu, but not in Punan Batu (Figs. S8D, S9D). However, we cannot know for certain whether these admixture connections represent links with local hunter-gatherers or the remnants of early MSEA ancestry in the overall Borneo gene-pool. Analysis of a wider range of northern and southeast agricultural Bornean populations (Figs. S8A-C, S9A-C) confirmed no direct ancestry contributions from the Punan Batu, though potential complexity in southern coastal East Asian ancestry related sources, and additionally revealed complexity in MSEA ancestry components.

We also generated qpGraphs including genotype data from a Mid Neolithic Suogang individual (Suogang-B1; Fig. S4). The result shows that the pre-Austronesian ancestry contribution towards the Punan predates the split between the Austronesian Kankanaey and the ancient Suogang individual. Finally, we performed the qpGraph analysis using the ancient historical North Bornean (Ma554) individual, as shown in Fig. S5. The connection of Ma554 to modern Austonesian groups is apparent, as is often observed with other Borneans. The Ma554 individual is closest to the Lundayeh (and Murut) group, as expected from the geographical proximity, indicating that the genetic segregation between Punan groups and the NE Bornean agriculturalist was still evident 400 years ago.

### Inferring Split Times

We performed the MSMC-IM^46^ analyses to infer split times between a pair of populations. MSMC-IM is an extension of the MSMC2. It fits a time-dependent migration model to the pairwise rate of coalescences based on estimates of coalescence rates within and across populations. The program requires phased whole-genome data as input files to the algorithm, thus, we were unable to infer Punan groups in this analysis as the data we generated from Punan is genome-wide SNP data. We then infer the split between Kankanaey and {Onge, Ami, Dusun, Maanyan} and between Maanyan and Dusun as well (Fig. 3), which have whole genome data, to get the sense of time from the split scenario inferred by the qpGraphs. The Kankanaey-Onge divergence serves as the upper bound for Punan split time inference, while the Kankanaey-Dusun and Kankanaey-Ami divergences serve as the lower bound, supporting the evidence using the ancient DNA data.

As inferred by the above analyses, Punan ancestry predates the Proto Austronesian/ancient coastal Southeastern Chinese ancestry (at least more than ~7,500 years ago), and hence its presence in the archipelago might be as old. To test possible connections between the Punan Batu and ancient hunter-gatherer on the neighboring island, Sulawesi, from the Leang Panninge site (~7,300 yBP) which has Papuan ancestry and Onge/Tianyuan ancestry,^5^ we further generated qpGraphs incorporating its genotype data (47,341 overlapping SNPs). This finds a weak signal of migration from Leang Panninge to Punan Batu (~0-2.1%; Fig. 4B), which is also tentatively supported by the D-statistics analysis (Fig. 4A, S6A). To confirm this signal, we used TreeMix v1.3^60^. We performed TreeMix analyses incorporating ancient DNA samples from the Leang Panninge site, which similarly visualize the migration node with a minor admixture proportion (1.05%) from Leang Panninge, but not the modern Papuan ancestry, to the Punan Batu. Together, these analyses highlight the possibility of further complexity in Punan ancestry related to the Leang Panninge sample.

## Supplementary Material

Suppelementary Material

## Data Availability

The new genotype data have been submitted at the European Genome-Phenome Archive (EGA), hosted by the EBI and CRG (accession number: EGAS00001004471). For consistency of process, all requests to access the sequences presented in this work are managed through the Data Access Committee of this official data repository. The main analysis code is publicly available (see Key Resources Table). Any additional information is available from the lead contact upon request.
