# Supplementary material for: Deep ancestry of Bornean hunter-gatherers supports long-term local ancestry dynamics
Source: Cell Rep. Author manuscript; Available in PMC 2025 Sep 11. (PMC7618103; doi:10.1016/j.celrep.2023.113346)
Supplement: Suppelementary Material [file EMS207970-supplement-Suppelementary_Material.pdf]

Supplementary Materials for

Deep ancestry of Bornean hunter-gatherers supports long-term local ancestry dynamics

Pradiptajati Kusuma*, Murray P. Cox, Graeme Barker, Herawati Sudoyo,
J. Stephen Lansing, Guy S. Jacobs*

*Corresponding authors

Supplementary Information include:

Supplementary Figures (Figs. S1 to S9)

Supplementary Tables (Table S1)

Supplementary Figures

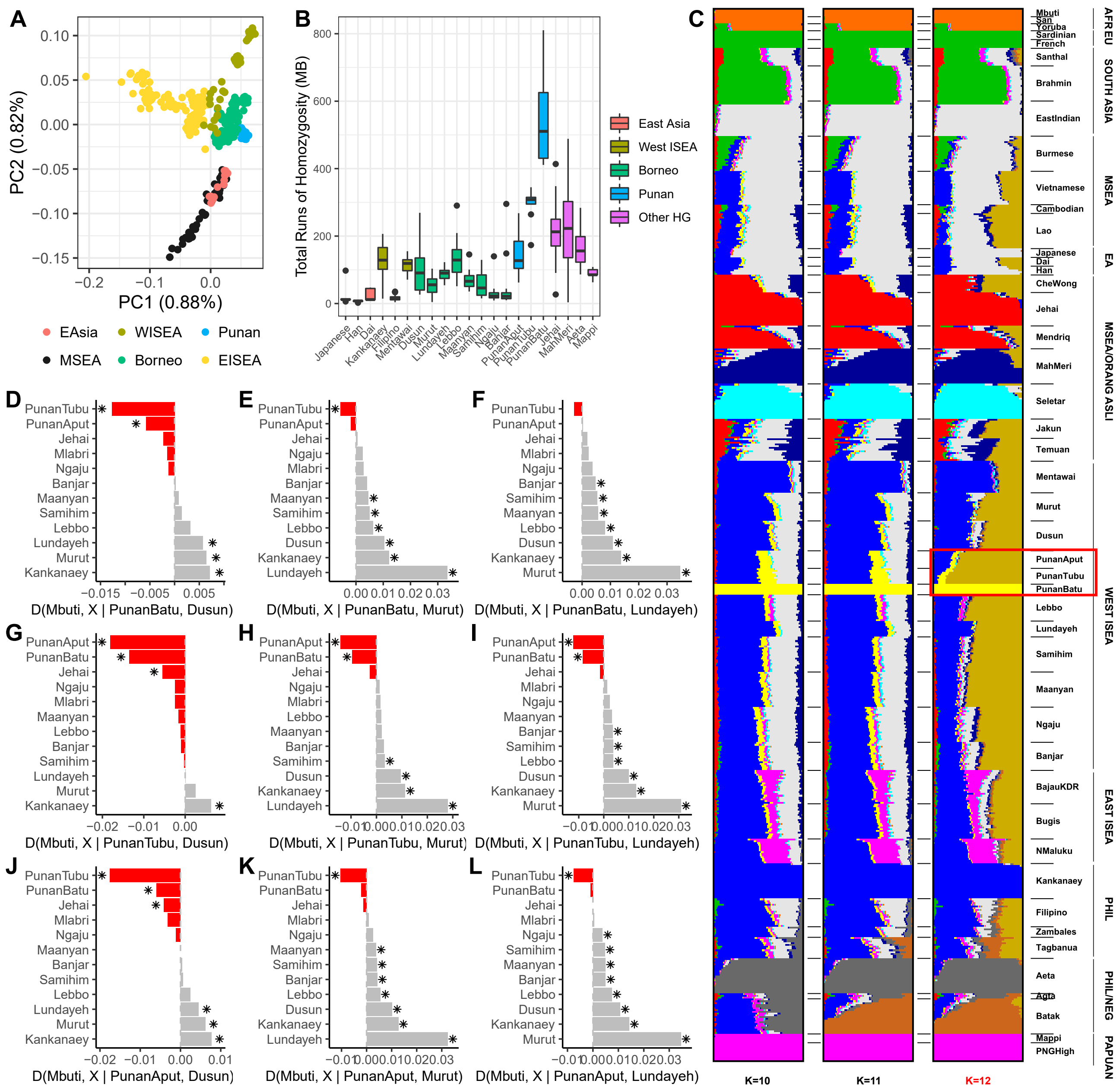

Fig. S1. (**A**) Principal Component Analysis on the regional level showing all Punan individuals cluster together, nearby but distinct from other Bornean groups. (**B**) Distribution of total per-sample runs of homozygosity (ROH) in representative Asian agricultural and traditionally hunter-gatherer groups. Traditionally hunter-gatherer groups, including the Punan, have relatively greater homozygosity than regional agriculturalists. (**C**) Admixture plot identifying the distribution of ancestry clusters among Asian and representative global populations. Bornean ancestry is complex, and includes a Punan-specific component that is maximised in the Punan Batu when K = 12 (most statistically supported number of clusters). (**D-L**) D-statistics assessing relationships between the Punan populations (Punan Batu, Punan Tubu and Punan Aput) and representative NE Bornean agriculturalists (Dusun, Murut and Lundayeh). Punan populations tend to show excess similarity to one another compared to other Bornean groups, supporting a cohesive Punan genetic clade.

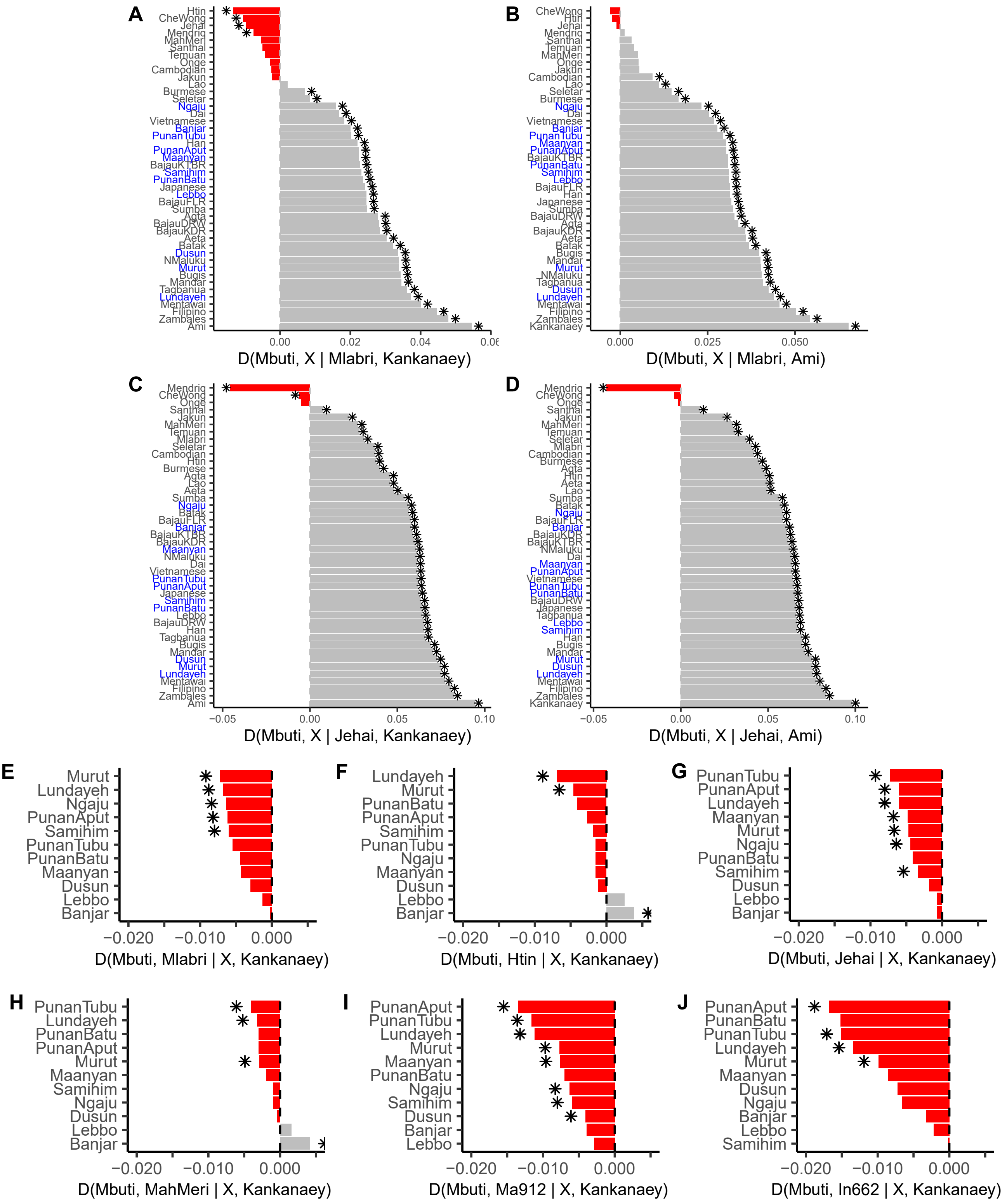

Fig. S2. (**A-D**) D-statistics assessing relationship between Mainland Southeast Asians vs. "Austronesian" proxies. All island Southeast Asians, including the Borneans (in blue) show positive D-statistics reflecting excess genetic similarity to the Kankanaey and Ami compared to MSEA groups. (**E-J**) We further conduct D-statistics assessing the relationship between Bornean and MSEA populations, revealing generally closer connections between MSEA groups and modern Borneans than the Kankanaey. Negative D-statistics correspond to excess similarity of MSEA groups to Bornean (X) populations, while positive D-statistics correspond to excess similarity to MSEA groups to the Kankanaey.

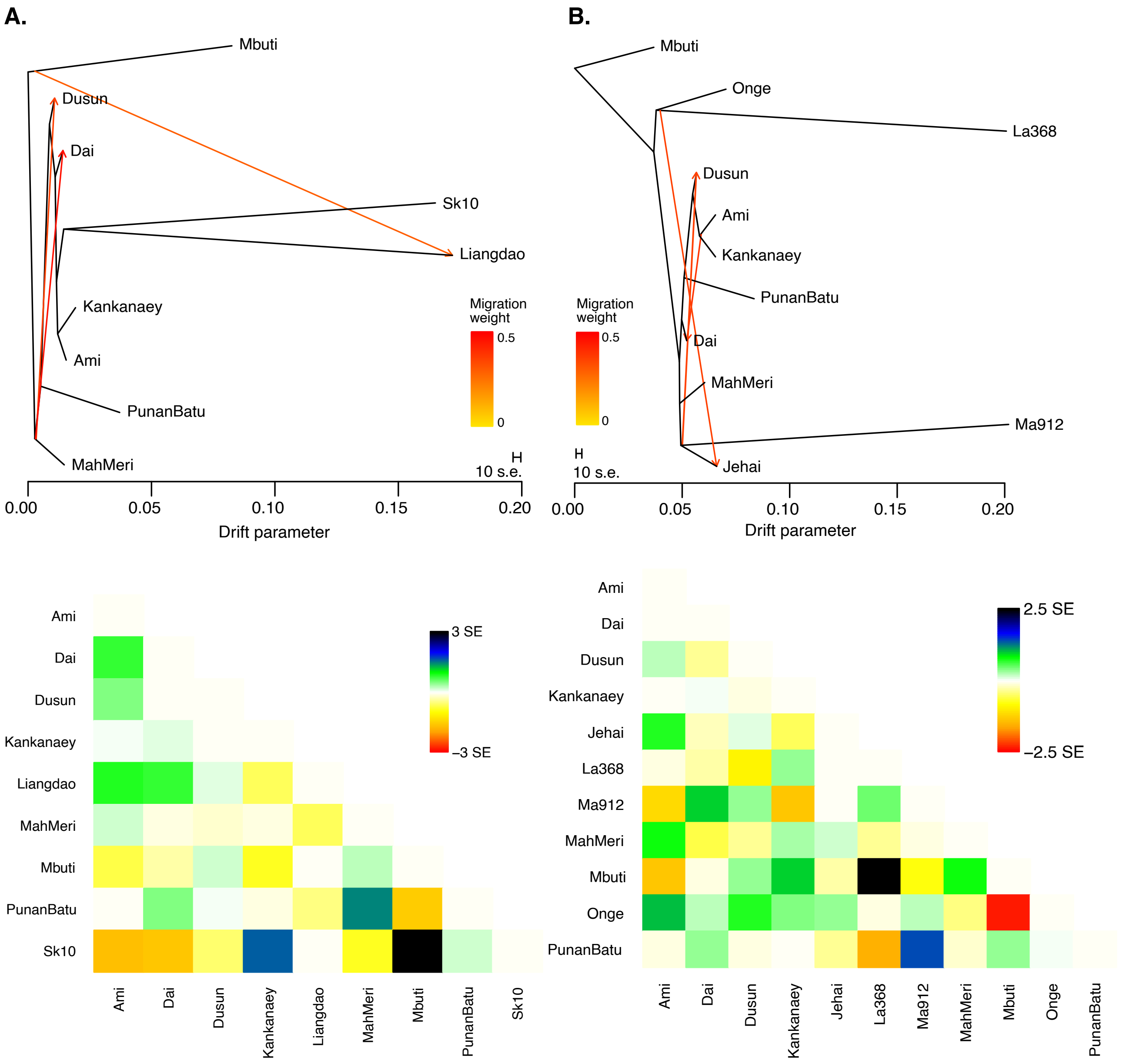

Fig. S3. TreeMix analyses with ancient East Asian (Liangdao2) and Lapita (Sk-10) individuals (**A**), and Early (La368) and Late Neolithic (Ma912) individuals (**B**) from Mainland Southeast Asia, confirming the qpGraph topologies in Fig. 2A-B.

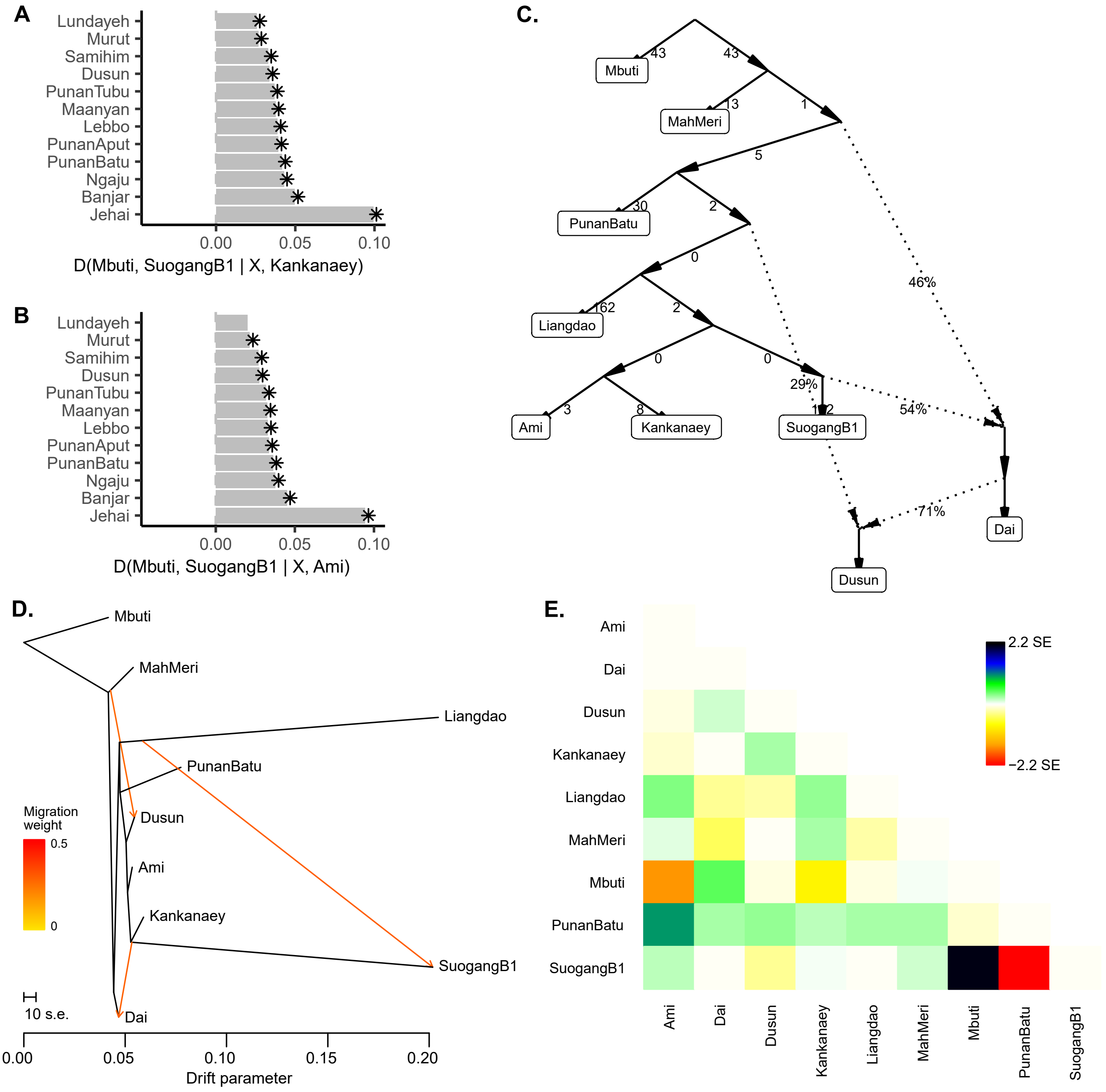

Fig. S4. (**A-B**) D-statistics assessing relationship between Bornean populations and ancient individuals (SuogangB1, mid-Neolitic "coastal southern East Asia ancestry"), relative to modern Austronesian proxy populations. Negative D-statistics correspond to excess similarity of the ancient samples to Bornean or MSEA (X) populations, while positive D-statistics correspond to excess similarity to the ancient samples to the Kankanaey/Ami. (**C**) A qpGraph (worst |Z| = 2.232) (**D-E**) and TreeMix exploring the position of the Punan Batu when an ancient mid-Neolithic coastal southern East Asia ancestry sample (SuogangB1) is included.

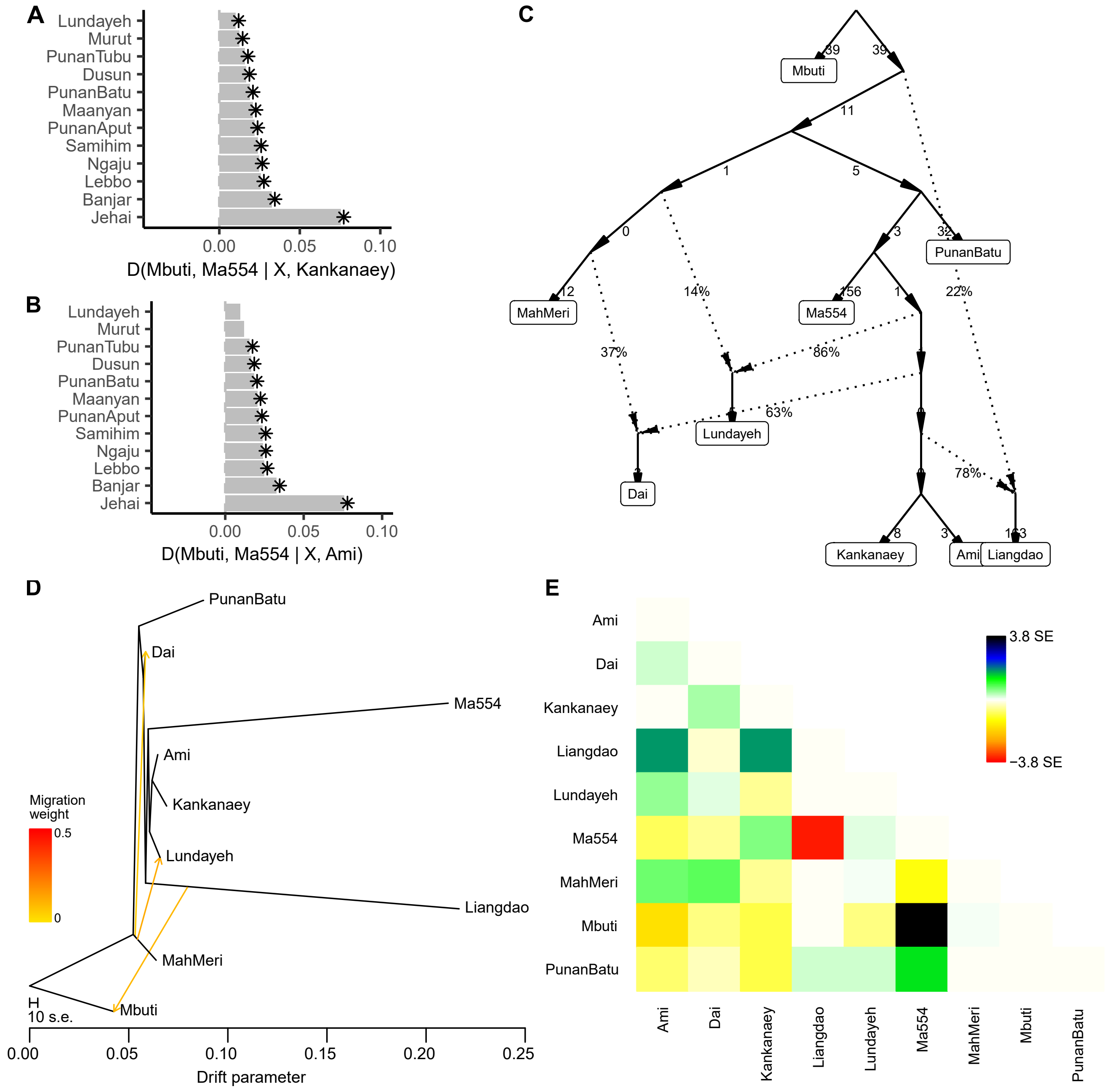

Fig. S5. (**A-B**) D-statistics assessing relationship between Bornean populations and ancient individuals (Ma554, historic north Bornean), relative to modern Austronesian proxy populations. Negative D-statistics correspond to excess similarity of the ancient samples to Bornean or MSEA (X) populations, while positive D-statistics correspond to excess similarity to the ancient samples to the Kankanaey/Ami. (**C**) A qpGraph (worst $|Z| = 2.447$) (**D-E**) and TreeMix exploring the position of the Punan Batu when an ancient historical Northeast Bornean sample (Ma554) is included.

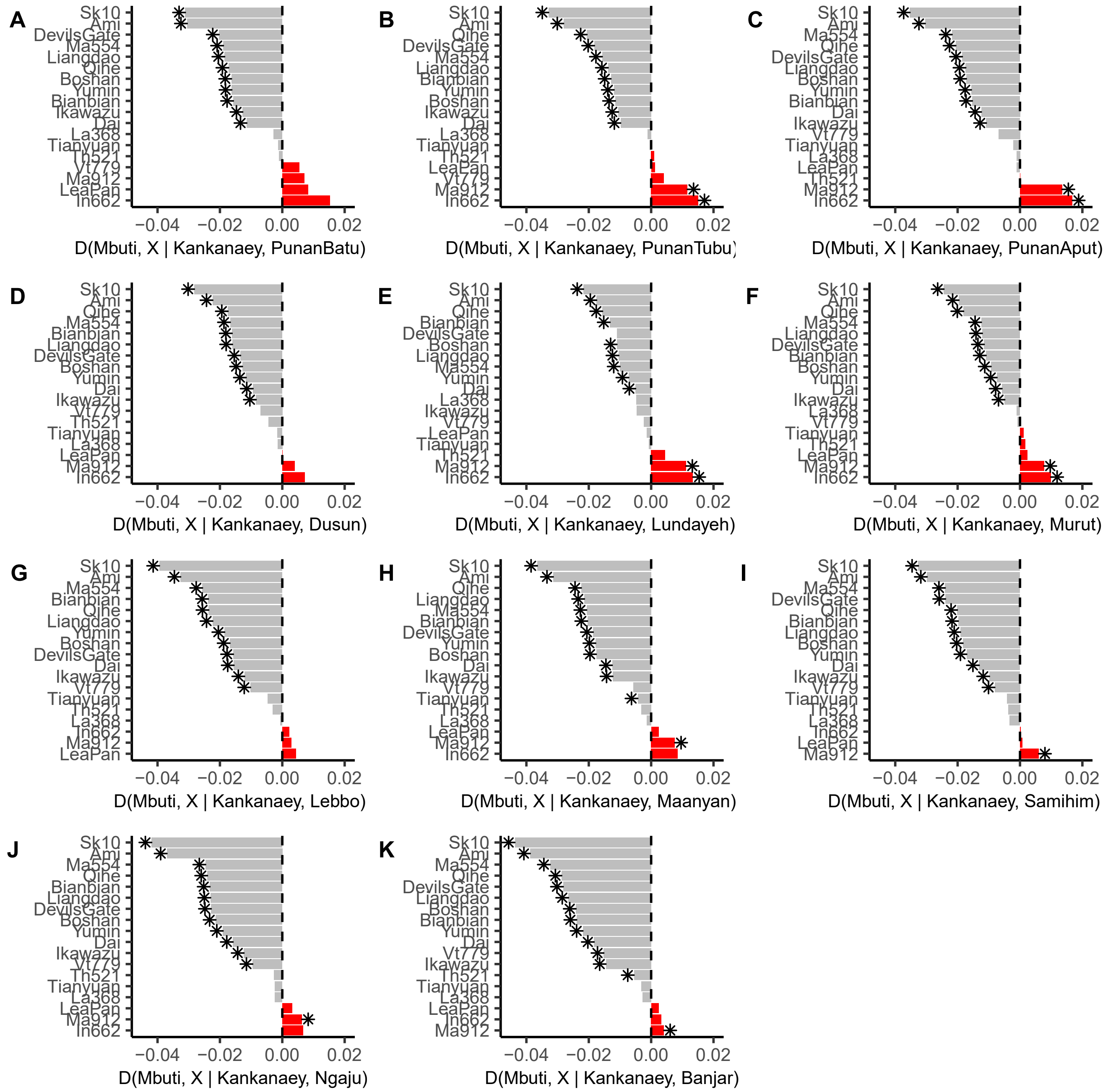

Fig. S6. D-statistics assessing relationship between East and Southeast Asian ancient samples (and the Dai and Ami) and Bornean populations, relative to a modern Austonesian proxy (Kankanaey). Negatives D-statistics indicate more similarity to the Kankanaey while positive D-statistics indicated more similarity to Bornean groups.

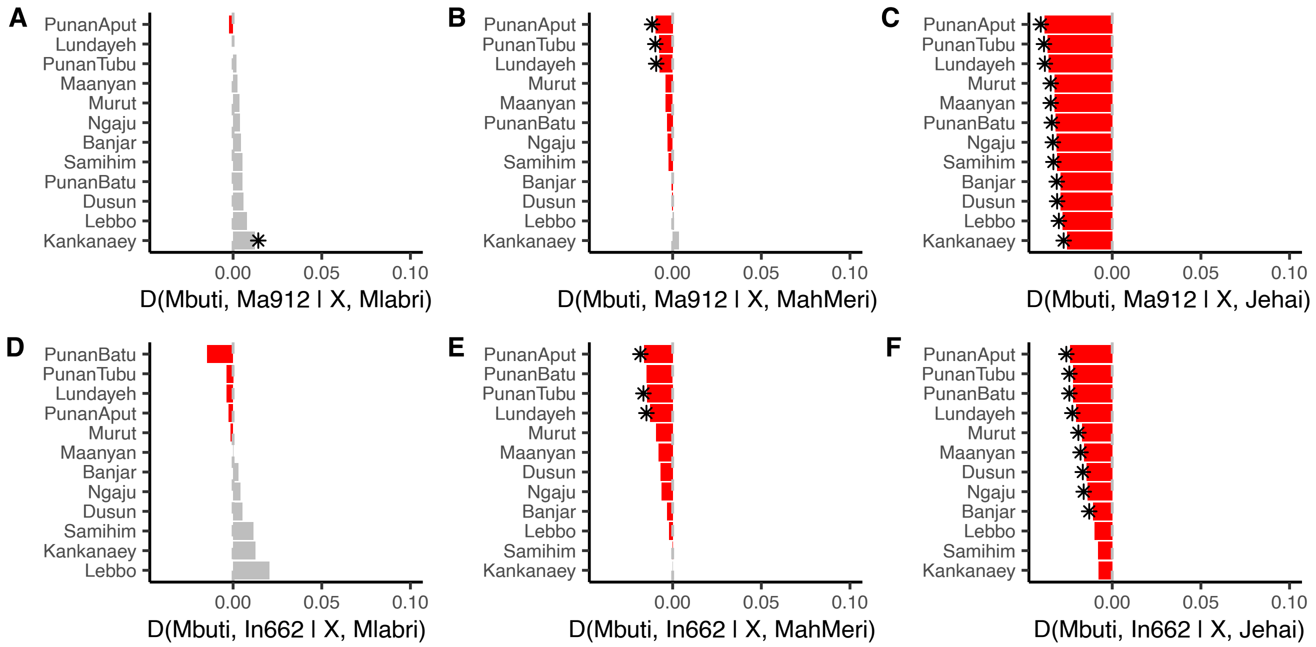

Fig. S7. D-statistics assessing relationship between Bornean populations and ancient individuals (Ma912, Late Neolithic mainland Southeast Asian; and In662, Late Neolithic northern highland Sumatran), relative to mainland Southeast Asian Orang Asli populations. Negative D-statistics correspond to more similarity of Ma912/In552 to Bornean (X) populations, while positive D-statistics correspond to more similarity to MSEA populations.

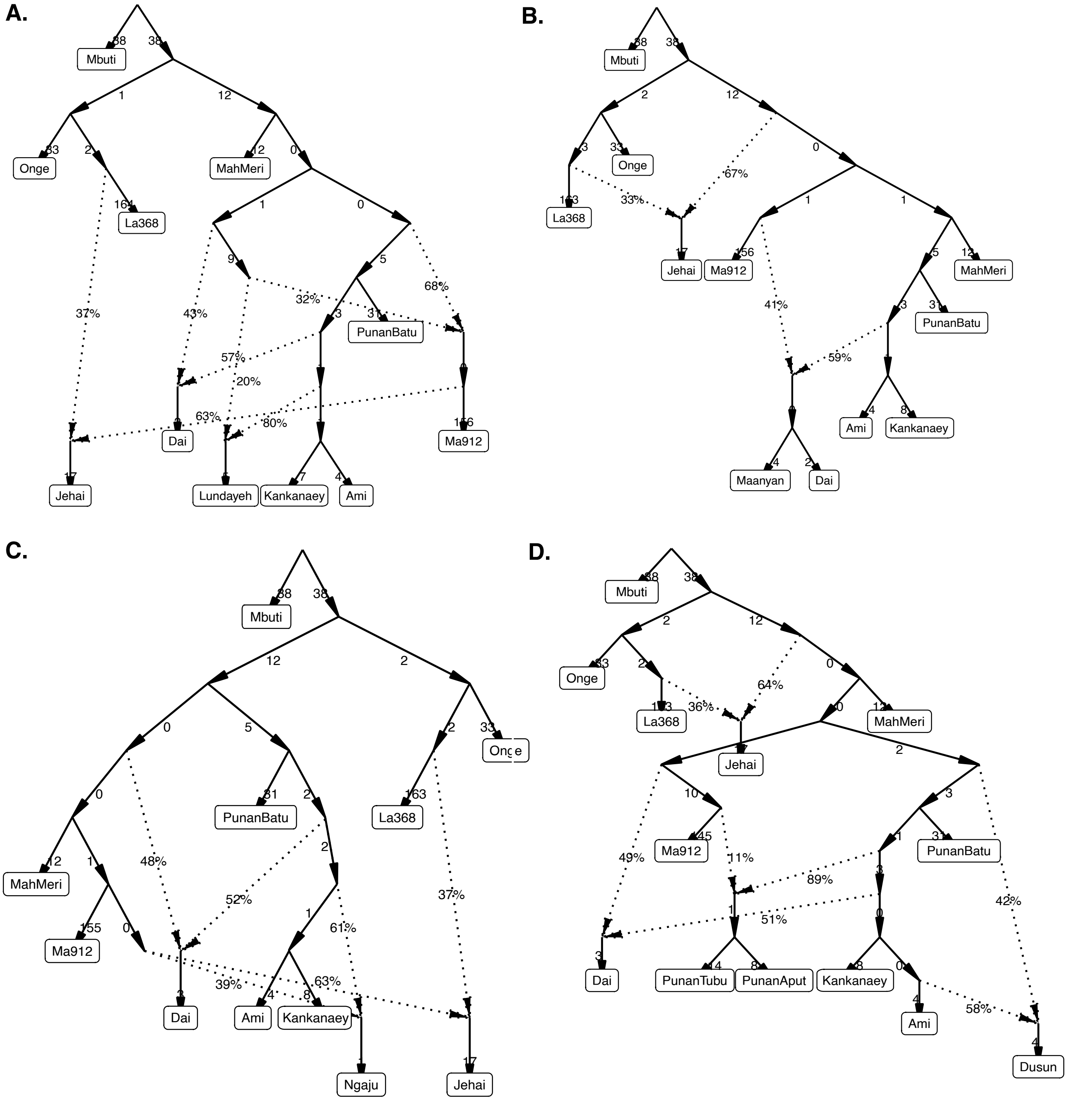

Fig. S8. A qpGraph exploring the complex ancestry of Borneo, by considering (**A**) a northeastern Borneo population (the Lundayeh; worst |Z| = 2.834), (**B**) southern Borneo populations (the Maanyan; worst |Z| = 2.625) and (**C**) the Ngaju (worst |Z| = 2.662), also (**D**) exploring the demographic and admixture history of Punan Batu with Punan Tubu and Punan Aput (worst |Z| = 2.889) in the model.

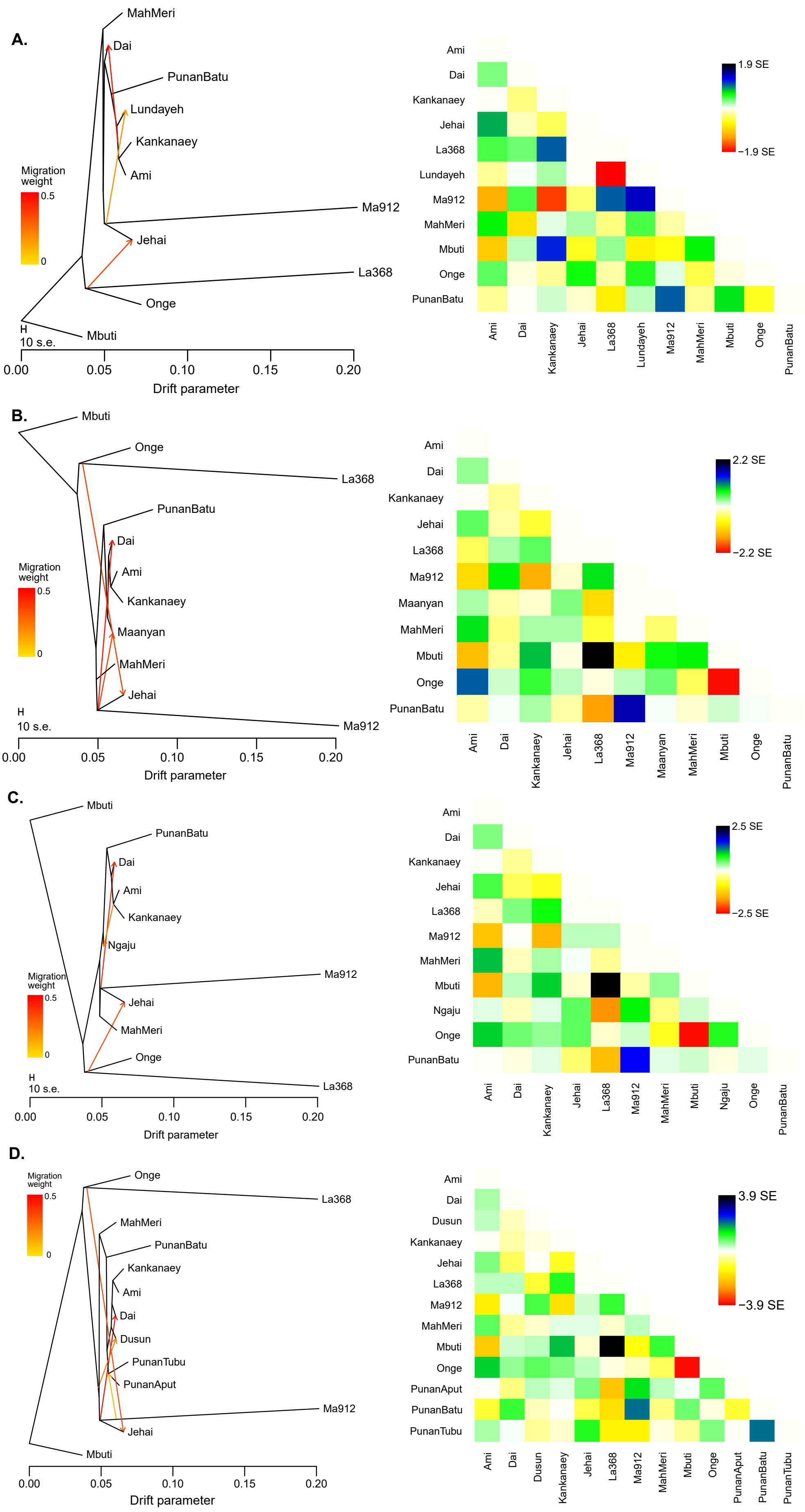

Fig. S9. A TreeMix exploring the complex ancestry of Borneo, by considering (**A**) a northeastern Borneo population (the Lundayeh), (**B**) southern Borneo populations (the Maanyan) and (**C**) the Ngaju, also (**D**) exploring the demographic and admixture history of Punan Batu with Punan Tubu and Punan Aput in the model.

Population	n	Source
Mbuti	4	Li et al. 2008
San	4	Li et al. 2008
Yoruba	4	Li et al. 2008
Sardinian	5	Li et al. 2008
French	5	Li et al. 2008
Santhal	10	Tatte et al. 2019
Brahmin	20	Morseburg et al. 2015
East Indian	20	Tatte et al. 2019
Burmese	20	Morseburg et al. 2015
Vietnamese	19	Morseburg et al. 2015
Cambodian	5	Li et al. 2008
Lao	20	Tatte et al. 2019
Japanese	5	Li et al. 2008
Dai	5	Li et al. 2008
Han	5	Li et al. 2008
Che Wong	10	Aghakhanian et al. 2015
Jehai	19	Aghakhanian et al. 2015
Mendriq	13	Aghakhanian et al. 2015
Mah Meri	20	Aghakhanian et al. 2015
Seletar	20	Aghakhanian et al. 2015
Jakun	11	Aghakhanian et al. 2015
Temuan	13	Aghakhanian et al. 2015
Mentawai	18	New Data
Murut	16	Morseburg et al. 2015
Dusun	17	Morseburg et al. 2015
Lundayeh	9	New Data
Punan Aput	10	New Data

Population	n	Source
Punan Tubu	9	New Data
Punan Batu	12	Lansing et al. 2022
Lebbo	15	Morseburg et al. 2015
Maanyan	20	Kusuma et al. 2016
Samihim	20	Kusuma et al. 2017
Ngaju	20	Kusuma et al. 2016
Banjar	16	Kusuma et al. 2016
BajauDRW	16	Kusuma et al. 2017
BajauKTBR	20	Kusuma et al. 2017
BajauKDR	20	Morseburg et al. 2015
BajauFLR	8	Morseburg et al. 2015
Bugis	20	Kusuma et al. 2017
Mandar	20	Kusuma et al. 2017
N. Maluku	14	Kusuma et al. 2017
Sumba	20	Cox et al. 2016
Kankanaey	20	Morseburg et al. 2015
Filipino	16	Morseburg et al. 2015
Zambales	6	Migliano et al. 2013
Tagbanua	12	Migliano et al. 2013
Aeta	20	Migliano et al. 2013
Agta	3	Migliano et al. 2013
Batak	20	Migliano et al. 2013
Mappi	5	New Data
Koinambe	10	Migliano et al. 2013
Kosipe	5	Migliano et al. 2013
PNG Highland	10	Li et al. 2008

Table S1. List of populations

References for Supplementary Information

1. Aghakhanian, F., Yunus, Y., Naidu, R., Jinam, T., Manica, A., Hoh, B.P., and Phipps, M.E. (2015). Unravelling the genetic history of Negritos and indigenous populations of Southeast Asia. Genome Biol. Evol. 7, 1206-1215. 10.1093/gbe/evv065.

2. Cox, M.P., Hudjashov, G., Sim, A., Savina, O., Karafet, T.M., Sudoyo, H., and Lansing, J.S. (2016). Small traditional human communities sustain genomic diversity over microgeographic scales despite linguistic isolation. Mol. Biol. Evol. 33, 2273-2284. 10.1093/molbev/msw099.

3. Kusuma, P., Brucato, N., Cox, M.P., Pierron, D., Razafindrazaka, H., Adelaar, A., Sudoyo, H., Letellier, T., and Ricaut, F-X. (2016). Contrasting linguistic and genetic origins of the Asian source populations of Malagasy. Sci. Rep. 6, 26066. 10.1038/srep26066.

4. Kusuma, P., Brucato, N., Cox, M.P., Letellier, T., Manan, A., Nuraini, C., Grangé, P., Sudoyo, H., and Ricaut, F-X. (2017). The last sea nomads of the Indonesian archipelago: genomic origins and dispersal. Eur. J. Hum. Genet. 25, 1004-1010. 10.1038/ejhg.2017.88.

5. Lansing, J.S., Jacobs, G.S., Downey, S., Norquest, P., Cox, M.P., Kuhn, S., Miller, J., Malik, S.G., Sudoyo, H., and Kusuma, P. (2022). Deep ancestry of collapsing networks of nomadic hunter–gatherers in Borneo. Evol. Hum. Sci. 4: E9. 10.1017/ehs.2022.3

6. Li, J.Z., Absher, D.M., Tang, H., Southwick, A.M., Casto, A.M., Ramachandran, S., Cann, H.M., Barsh, G.S., Feldman, M., Cavalli-Sforza, L.L., and Myers, R.M. (2008). Worldwide human relationships inferred from genome-wide patterns of variation. Science. 319, 1100-1104. 10.1126/science.1153717.

7. Migliano, A.B., Romero, I.G., Metspalu, M., Leavesley, M., Pagani, L., Antao, T., Huang, D.W., Sherman, B.T., Siddle, K., Scholes, C., Hudjashov, G., Kaitokai, E., Babalu, A., Belatti, M., Cagan, A., Hopkinshaw, B., Shaw, C., Nelis, M., Metspalu, E., Mägi, R., Lempicki, R.A., Villems, R., Lahr, M.M., and Kivisild, T. (2013). Evolution of the pygmy phenotype: evidence of positive selection fro genome-wide scans in African, Asian, and Melanesian pygmies. Hum. Biol. 85, 251-284. 10.3378/027.085.0313.

8. Mörseburg, A., Pagani, L., Ricaut, F-X., Yngvadottir, B., Harney, E., Castillo, C., Hoogervorst, T., Antao, T., Kusuma, P., Brucato, N., Cardona, A., Pierron, D., Letellier, T., Wee, J., Abdullah, S., Metspalu, M., and Kivisild, T. (2016). Multi-layered population structure in Island Southeast Asians. Eur. J. Hum. Genet. 24, 1605-1611. 10.1038/ejhg.2016.60.

9. Tätte, K., Pagani, L., Pathak, A.K., Kõks, S., Ho Duy, B., Ho, X.D., Sultana, G.N.N., Sharif, M.I., Asaduzzaman, M., Behar, D.M., Hadid, Y., Villems, R., Chaubey, G., Kivisild, T., and Metspalu, M. (2019). The genetic legacy of continental scale admixture in Indian Austroasiatic speakers. Sci. Rep. 9, 3818. 10.1038/s41598-019-40399-8.
